# Viral Prevalence in Wild Serval Population is Driven by Season and Sex

**DOI:** 10.1007/s10393-021-01533-z

**Published:** 2021-05-31

**Authors:** Daan J. E. Loock, Emilio Rendón-Franco, Samual T. Williams, Johan van Niekerk, Lourens H. Swanepoel

**Affiliations:** 1 Centre for Sustainable Agriculture, Faculty of Natural and Agricultural Sciences, University of the Free State, 205 Nelson Mandela Drive, Park West, Bloemfontein, 930 South Africa; 2 Departamento de Producción Agrícola y Animal, UAM-Unidad Xochimilco, Calzada del Hueso, Coyoacán, Ciudad de México, 04960 México; 3 Department of Zoology, School of Mathematical and Natural Sciences, University of Venda, Private Bag X5050, Thohoyandou, 0950 South Africa; 4 Department of Anthropology, Durham University, Durham, DH1 3LE UK; 5 Institute for Globally Distributed Open Research and Education (IGDORE), Göteborg, Sweden

**Keywords:** Carnivore, Felidae, Wildlife disease

## Abstract

One of the key factors influencing the population dynamics of threatened species such as felids is disease, but long-term studies of the factors influencing seroprevalence of wild felids are extremely rare, hindering conservation efforts. We set out to determine seroprevalence of six viral diseases (feline panleukopenia virus, feline leukemia virus, feline coronavirus, feline calicivirus, feline herpes virus, and feline immunodeficiency virus) among a population of serval (*Leptailurus serval*) with an extremely high density in South Africa. We captured 55 individuals over four years and screened blood samples for antibodies to each virus. We found that seroprevalence were high (ranging from 30.0% positive for a single virus to 1.8% positive for up to five viruses) and that seroprevalence was influenced by season and sex, but not body condition. We suggest further monitoring of this population and recommend that long-term studies are conducted for serval and other felids to determine whether these trends are representative on a broader scale.

**Supplementary Information:**

The online version contains supplementary material available at 10.1007/s10393-021-01533-z.

## Introduction

In wildlife populations, disease has many detrimental effects. For example, among feline viruses, feline coronavirus can cause chronic diarrhea associated with inappetence, weight loss, and severe immunosuppression (Kennedy et al. 2003). This causes increased mortality rates and reduced recruitment (e.g., higher abortion rates and deaths by secondary causes) (Kennedy et al. 2003; Bradley and Altizer 2007), which may lead to local population extinction (Franklin et al. 2008). Feline leukemia virus for example has been identified as the most prominent cause of mortality in an Iberian lynx (*Lynx pardinus*) population with different agents (such as canine parvovirus, *Toxoplasma gondii*) (Meli et al. 2010) that have the potential to provoke deaths in this carnivore population (Bradley and Altizer 2007). This may have a similar devastating effect on other carnivore populations that occur at a high density. Despite the importance of disease and the effects on wildlife, our understanding of mesocarnivore epidemiology is still hampered by a dearth of baseline data (Franklin et al. 2008; Munson et al. 2010). Natural hosts for diseases are poorly described for wildlife populations in general, and almost nothing is known in some geographical areas (Ostrowski et al. 2003). This is even true for relatively well-studied taxa such as felids, where several viral diseases are particularly important as they are often the cause of fatalities. The understanding of wild felid viral pathogens is therefore important to mesocarnivore conservation.

Several factors, both environmental and biological, are believed to affect disease prevalence and susceptibility. For example, one factor strongly associated with disease prevalence in mammals is their body condition index (BCI) (Renwick et al. 2007). The body condition index is a standardized noninvasive tool (Green 2001) to evaluate the fitness of an individual animal based on physical characteristics. Body condition index can be an indication of general fitness and fat deposits based on phenotypic observations (Schulte-Hostedde et al. 2005). BCI is therefore strongly related to reproduction and survival potential (Schulte-Hostedde et al. 2005). Furthermore, malnutrition, lameness, and respiratory infections all seem to directly affect body condition (Jakob et al. 1996; Renwick et al. 2007). BCI tends to be lower in infected individuals than in uninfected individuals (Fromont et al. 2000). A closely related factor, and often associated with BCI, affecting disease prevalence is season. For example, harsh weather conditions and temperature fluctuations will often result in poor nutrition (Smith et al. 2009) and low investment in reproduction, and weaken immune system (Dowell 2001; Pedersen 2009). These effects may cause undesirable consequences for wild felid species, so it is particularly important to protect native carnivores from these unpredictable outcomes (Ferreira and Funston 2010).

**Figure 1:**
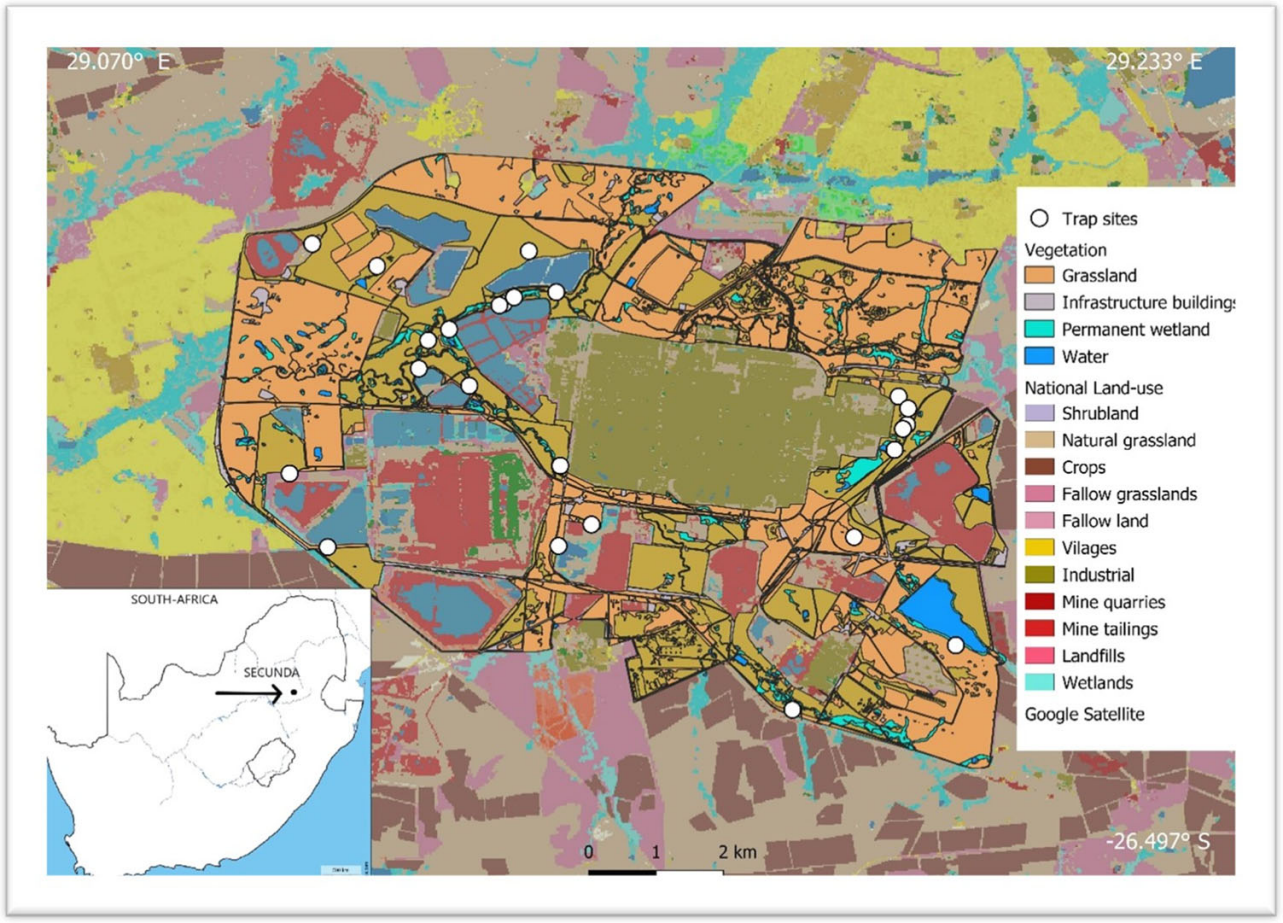
The study site in context of the larger surrounding landscape, highlighting human activities and land uses including settlements, agriculture, industry, and natural areas.

In this study, we focus on the viral prevalence of a population of serval (*Leptailurus serval*) inhabiting the natural areas surrounding an industrial petrochemical plant located in Secunda, Mpumalanga Province, South Africa (central coordinates 26°31′45.62′′ S, 29°10′31.55′′ E) (Loock et al. 2018). This population is a unique model system for disease ecology for several reasons. Firstly, the area has the highest serval density recorded (Loock et al. 2018) and such high densities can increase transmission rate and susceptibility to disease infection (Lindenfors et al. 2007). Secondly, even though the serval population seems to be stable through sampling periods, there are indications of population cycles through wet and dry seasons (Loock et al. 2018), which suggests population changes may be due to sources of mortality such as disease. Combined with high densities, the wet and dry seasons can add additional pressure, which could affect disease prevalence (Naidenko et al. 2014). Thirdly, the study area is embedded within a human-dominated matrix with varying levels of human activity, industrial contaminants, domestic animals (livestock and pets), and local native fauna (Fig. 1). We aim to provide the first long-term prevalence data for a range of viral diseases in this free-ranging serval population. Secondly, we evaluate body condition index (BCI) in combination with a suite of biological and environmental factors to investigate the factors associated with viral prevalence. We hypothesized that BCI will be affected by sex (higher in males compared to females) and season (lower in dry season). Furthermore, we hypothesized that feline virus prevalence will be affected by BCI (higher risk for low BCI scores), season (higher risk in wet season), sex (higher in females), and age (higher risk in older animals).

## Methods

### Study Site

Secunda Synfuels Operations Plant (a division of Sasol South Africa) is an industrial petrochemical plant located in Secunda, Mpumalanga Province, South Africa, which employs an estimated 12,500 permanent employees. The western boundary of the site is neighbored by an informal settlement (Embalenhle), with an estimated human population of approximately 147,122 (Matooane et al. 2011). The northern edge of the complex is adjacent to an industrial development, golf courses, and the Secunda town, with an estimated human density of 41,000 (Matooane et al. 2011).

The site has a rich diversity of mammal species, including 11 carnivore species; African clawless otter (*Aonyx capensis*), black backed jackal (*Canis mesomelas*), Cape fox (*Vulpes chama*), large grey mongoose (*Herpestes ichneumon*), serval, slender mongoose (*Galerella sanguinea*), small spotted genet (*Genetta genetta*), suricate (*Suricata suricatta*), water mongoose (*Atilax paludinosus*), white-tailed mongoose (*Ichneumia albicauda*), and yellow mongoose (*Cynictis penicillata*) (Emslie 2018).

### Live Trapping

Live trapping of serval formed part of a research project investigating wild serval spatial and disease ecology. We trapped serval during five sessions for a total of 477 trap nights between 2015 and 2018, using steel trap cages measuring 200 cm × 80 cm × 80 cm. We deployed cages at 41 different locations throughout the study site, which were selected based on information gained from camera trapping.

Captured servals were immobilized by a qualified veterinarian, using standard immobilization protocols for felids (Morris 2001). Blood samples were collected during the deployment of GPS collars, microchips, and the collection of body measurements including age, sex, and weight. Servals were classified as adults (body mass 10.0 kg or more), subadults: (7.0 kg to 9.9 kg), and juveniles: (< 7.0 kg) (Fromont et al. 2000). Samples from all 55 animals were screened for viral antigen or antibodies.

### Blood Sample Collection and Analysis

Blood samples were collected from the jugular veins using BD Vacutainer® tubes containing a clot activator and were centrifuged for ten minutes at 1500 revolutions per minute, after standing for approximately an hour after collection. All serum samples were submitted to the laboratory for analysis within 12 h after collection. IDEXX Laboratories located in Johannesburg, South Africa (SANAS accredited veterinary laboratory number V0040), were contracted to analyze the blood samples throughout the study.

### Viral Screening

Samples were screened for antibodies to the following viral pathogens: feline calicivirus (FCV), feline coronavirus (FCoV), feline herpesvirus (FHV), feline panleukopenia virus (FPLV), feline immunodeficiency virus (FIV), and for viral proteins for feline leukemia virus (FeLV). The indirect immunofluorescent antibody assay (IFA, IDEXX®) was used for FCV, FCoV, FHV and FPLV, and enzyme-linked immunosorbent assay (ELISA, IDEXX®) was used for FIV and FeLV. For FIV, ELISA IDEXX ®, even when there are different species-specific FIV, the test has showed concordance with some FIV from wildcats with just slightly less sensitivity (Brown et al. 2010).

### Calculation of Body Condition Index

We calculated serval body condition index (BCI), a proxy for body fat (Labocha et al. 2014), using the log body mass/log body length ratio. Body mass was the total body weight (kg) of captured animals, and body length was measured from the tip of the nose to the base of the tail (cm). Although the use of ratio indices has limitations when comparing between species, they are adequate for intraspecific comparisons (Jakob et al. 1996; Fromont et al. 2000).

### Data Analysis

Our modeling framework was underpinned by an information theoretic approach, which allowed us to test several biologically plausible hypotheses, using multiple linear regression (Burnham and Anderson 2002). To investigate the variation in BCI, we fitted linear models to test several hypotheses. First, we hypothesized that sex influences BCI since male animals generally have higher BCI compared to females (Windberg et al. 1991; Pulliainen et al. 1995). Further, we let BCI vary by season since seasonal effects such as ambient conditions and prey availability may influence BCI (Windberg et al. 1991; Pulliainen et al. 1995). We also fitted models including both season and sex, as well as a season and sex interaction models, since females lactating in dry season will incur higher metabolic costs (Windberg et al. 1991).

We further hypothesized that BCI, season, sex, and age of serval could affect the prevalence of the diseases tested. We used generalized linear models with a log link function and binomial distribution to estimate the effect of BCI, sex, age, season, sex + age, and sex + season on the prevalence (Luo et al. 2014). We restricted the model fitting to these variable combinations to avoid over parametrization of models. Relative risk can be seen as a ratio of success probability in a specific group compared to another (Luo et al. 2014). We fitted a log-binomial model for each disease separately (FCV, FHV, FCoV, FPLV, and FIV) and ranked models according to Akaike’s information criterion corrected for small sample sizes (Akaike 1974; Luo et al. 2014). Since we had a small number of animals recaptured on successive occasions, we removed re-captured animals from analysis to avoid pseudo-replication. Model fit was evaluated for the most parsimonious model using the Hosmer–Lemeshow goodness of fit test and visual inspection of the residual plots. Log-binomial regression models were fitted by maximizing the likelihood based on the expectation–maximization (EM) algorithm, which seems to improve likelihood estimation, using the *logbin* package (Donoghoe and Marschner 2018) in R version 3.6.1 (Pinheiro et al. 2019). Models within a ΔAICc of 2 were considered to have equal support. We further assessed variable importance using two methods. First, variables falling within the ΔAICc of 2 of the top models were further assessed by sequential likelihood ratio tests. We dropped variables if the likelihood ratio suggested that variable reduced model fit. Secondly, we assessed the overlap in confidence intervals for predicted variables and dropped variables if significant overlap in confidence intervals occurred (Cumming 2009).

We estimated viral prevalence as the proportion of animals that tested positive for antibodies to pathogens (except FeLV, with all servals testing negative). Confidence intervals for pathogen prevalence were estimated using the Wald interval (normal approximation interval) using the *prevalence* R package (Devleesschauwer et al. 2014). All figures were prepared using *ggplot2* (Wickham 2009).

## Results

During the study period, 55 animals were captured (including recaptures), which consisted of 34 adults (21 males and 13 females), 20 subadults (5 males and 15 females), and one juvenile (a female). Most animals were trapped in the wet season (47 animals), with only 8 individuals captured in the dry season (Fig. 1S). From these captures, several animals were trapped on more than one occasion. Three males were captured twice, two females were also captured twice, and one male was captured on three occasions.

### Body Condition Index

**Figure 2:**
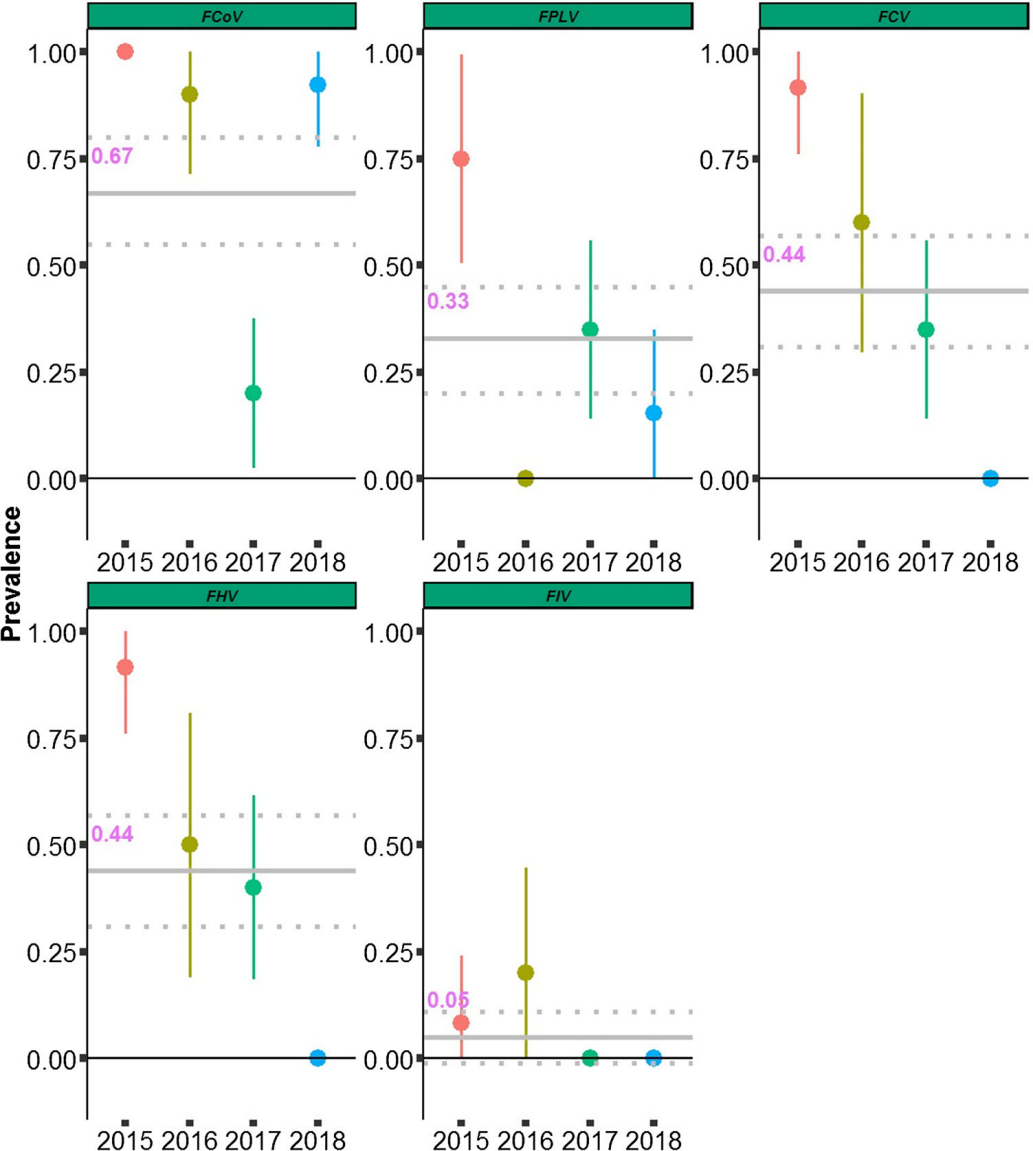
Annual variation in viral prevalence for five viruses tested (FCoV, FPLV, FCV, FHV, and FIV). Solid grey horizontal line represents the mean prevalence over study period and dotted lines 95% confidence interval; vertical lines represent 95% confidence interval for yearly prevalence rates.

Due to uncertainty in age classification of subadults, we restricted BCI analysis to adult serval. We found support that the body condition index (BCI) was affected by sex of serval (Fig. 2S; combined model weight of < 2 ∆ AICc = 0.78), with little support for seasons (Fig. 2S), or the season*sex interaction (Fig. 2S). Male serval had a higher BCI (mean = 0.57) compared to female serval (mean = 0.54; Fig. 2S).

### Viral Screening

**Figure 3:**
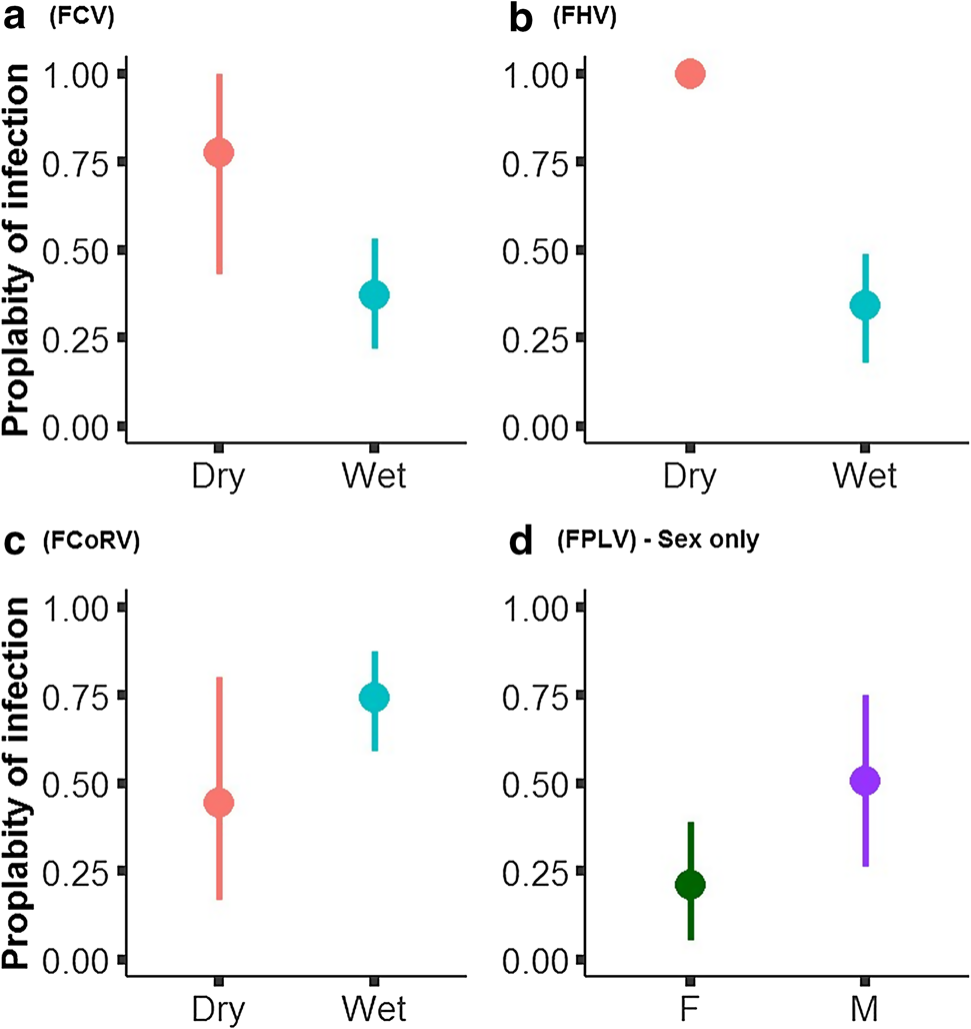
The probability of pathogen infection, influenced by season for **a** FCV, **b** FHV, **c** FCoV, and **d** FPLV affected by sex only.

Of the 55 individual servals we screened for viral diseases, antibodies were detected for FCV, FCoV, FHV, FPLV, and FIV (Fig. 2). All samples screened against FeLV resulted as FeLV-antigen negative. Prevalence for diseases screened varied over the sampling period (Fig. 2). Two viral diseases (FCoV and FPVL) showed declines and increases over study period (Fig. 2), while FCV and FHV showed sharp decline since first sampling (Fig. 2). Mean prevalence over the 4-year sampling period was highest for FCoV (mean = 67%; 95% CI: 55–80%), followed by FCV and FHV (mean = 44%, 95% CI: 31–57%, respectively), and FPLV (mean = 33%; 95% CI: 20–45%) (Fig. 2). FIV had the lowest prevalence of the 4-year sampling period mean = 5% (95% CI: 0–11%) (Fig. 2). Ten animals (18% of those screened) had no antibodies for any of the viruses tested, 17 individuals (30%) were positive for only one virus, eight individuals (15%) tested positive for two viruses, eight individuals (15%) tested positive for 3 viruses, 11 individuals (20%) tested positive for four viruses, and one animal (2%) tested positive for 5 viruses (Fig. 3S).

### Variables Affecting Prevalence

While there was model uncertainty in variables affecting probability of viral prevalence (Table 1; Table 1S), there was support for season as a main factor affecting probability of prevalence for FCoV, FCV, and FHV (Table 1; Table 1S). Likelihood ratio tests suggested that dropping sex from FCoV (*p* = 0.465) and FCV (*p* = 0.0515) improved model fit, which was confirmed with high overlap in confidence intervals between sexes in models which included sex and season affecting prevalence (Fig. 4S). Seroprevalence during the wet season was approximately half during the dry season for both FCV (wet season relative risk = 0.51) and FHV (wet season relative risk = 0.34; Fig. 3). In contrast, for FCoV, the prevalence was about double in the wet season compared to dry (wet season relative risk = 1.6; Fig. 3). For FPLV, there was support for both sex and season affecting prevalence (Table 1; Table 1S); however, likelihood ratio tests suggested that dropping season improved model fit (*p* = 0.202), with further support from large overlap in 95% confidence intervals for prevalence between seasons (Fig. 4S). As such, we retained only sex as a factor affecting FPLV prevalence where males had double the infection probability of females (male relative risk = 2.25; Fig. 3). For most of the viruses tested, we did not find support for BCI affecting the prevalence except for FCoV, where BCI seems to play a minor role (model weight of < 2 ∆AICc = 0.16; Table 1; Table 1S). Interestingly, the prevalence seems to increase with higher BCI scores (Fig. 5S).

**Table 1:** Summary of the Model Results and Ranking for the Various Variables Affecting the Different Pathogens Probability of Infection. Summary is Restricted to Models with ΔAICc of < 2, Full Model

	AICc	Delta	ModelLik	ModelWt
Disease—FCoV				
FCoV ~ season	56.56	0	1	0.39
FCoV ~ sex + season	58.34	1.77	0.41	0.16
FCoV ~ BCI	58.38	1.82	0.4	0.16
Disease—FPLV				
FPLV ~ sex	53.83	0	1	0.33
FPLV ~ sex + season	54.52	0.7	0.71	0.23
FPLV ~ season	55.75	1.92	0.38	0.13
Disease—FCV				
FCV ~ season	58.54	0	1	0.31
FCV ~ season + sex	59.54	1	0.61	0.19
FCV ~ sex	59.89	1.35	0.51	0.16
FCV ~ sex + season	60.47	1.94	0.38	0.12
Disease—FHV				
FHV ~ season	49.3	0	1	0.71

Results Can be Seen in Table 1S.

## Discussion

Approximately 82% of the servals screened over the course of the study tested positive for at least one of the five viruses included. This high prevalence, combined with large annual variation in serval population density (Loock et al. 2018), could suggest that viruses are probably an important factor in serval population dynamics at Secunda. Prevalence was high for most viruses, except for the retroviruses (FIV and FeLV). FIV prevalence could be underestimated because ELISA IDEXX® showed lower sensitivity for FIV belonging to wildcats of the Felis group (Brown et al. 2010). However, low FIV prevalence is common among wildcat, as we explain below. The prevalence of FCV, FCoV, FHV, and FPLV viruses was higher in our study than for most other wild African felids, except for some specific populations of lions in Tanzania and Uganda (Hoffman-Lehmann et al. 1996; Driciru et al. 2006), and caracals (*Caracal caracal*) in Namibia (Thalwitzer et al. 2010) although this study had a small sample size of 3 individuals. Virus prevalence often tends to be density dependent, so one possible explanation for this high prevalence in this study is the high density of this serval population (76.2–101.1 serval/ 100 km^2^) (Loock et al. 2018).

Retroviruses have low prevalence among the study population. This is similar to other African wild felid populations, where FIV has low prevalence, and there is no evidence of FeLV (Lutz et al. 1992; Kennedy et al. 2003; Ostrowski et al. 2003; Heddergott et al. 2018). The exceptions to this are lions with high prevalence of FIV (Hoffman-Lehmann et al. 1996; Brown et al. 1993; Driciru et al. 2006; Ramsauer et al. 2007) and African wild cats (*Felis silvestris lybica*) with evidence of FeLV infection (Ostrowski et al. 2003). In contrast, for domestic cats FeLV is usually endemic (Oguzoglu et al. 2013; Muchaamba et al. 2014), which can lead to wild felid infections which are usually naive to the virus (Muchaamba et al. 2014). Continuous monitoring for FeLV remains important since the study area is surrounded by human settlements which are likely to have high numbers of domestic cats. Spillover of FeLV from domestic cats to serval remains a possibility, and FeLV transmission from domestic cats remains a serious threat for other wild cat species (Meli et al. 2010; Brown et al. 2018; Chiu et al. 2019). The spillover risk poses the possibility for closely related strains of pathogens to simultaneously be transmitted to other susceptible feline species (Kellner et al. 2018). Other external factors may also influence pathogen transmission such as seasonal variation affecting host susceptibility (for instance due to changing atmospheric conditions) (Dowell 2001), host behavior may change, and photoperiod-driven changes influencing physiological changes in mammalian species (Dowell 2001).

The strongest driver of viral infection in our analysis was season. There are no data available on the seasonal effects of viral infection prevalence among African felids, but in domestic cats in shelters, season influences the prevalence of FHV and FCV (Zicola et al. 2009; Meli et al. 2010). For FHV prevalence peaked in spring and autumn, and FCV infections peaked in winter and spring (Zicola et al. 2009; Meli et al. 2010), which may be related to the parturition period (Zicola et al. 2009; Meli et al. 2010). This is supported by (Goller et al. 2013) who demonstrated the importance of juvenile spotted hyenas (*Crocuta crocuta*) for the maintenance of coronavirus. For serval, the highest prevalence for FHV and FCV was in the dry season (winter-spring), which corresponds with the pregnant-newborn season for serval (Smithers 1978). However, other seasonal variables should be considered, such as rainfall, temperature, and radiation, which directly affect the permanence of the virus in the environment. Times of increased physical contact between servals, for example, during mating season, could also play an important role.

Even though sex emerged as an important variable affecting pathogen infection, there was much uncertainly and overlap in infection probability between sexes. Sex was an important variable only for the prevalence of FPLV. This effect of sex on feline viral diseases has been explored for lion, cheetahs (*Acinonyx jubatus*), wildcat and sand cat (*Felis margarita*), although no clear relationship emerged (Hoffman-Lehmann et al. 1996; Ostrowski et al. 2003; Munson et al. 2004; Pomerantz et al. 2016). The only felid with clear effects of sex on pathogen infection was cheetah, where FHV was higher for males and FCV higher for females, although these results were not based on robust statistical analysis (Munson et al. 2004).

One factor that did not seem to be especially important in influencing the prevalence of most viruses was BCI. Nonetheless, body condition emerged as a driver for FCoV, but in an unexpected manner, with animals with a higher BCI being more likely to test positive for antibodies to FCoV. A possible explanation for this is that animals with antibodies are those who survived a prior infection (possibly due in part to their better body condition) and develop a serological immune response against the virus. We did, however, find support for the hypothesis that BCI would be greater among adult males than adult female serval. This concurs with findings in other species, for example, two thirds of male Eurasian lynx (*Lynx lynx*) in Finland had greater BCI than females (Pulliainen et al. 1995). Coyotes (*Canis latrans*) in Texas, USA, with a high population density also showed a similar trend, with males having a 17% higher BCI than females (Windberg et al. 1991). This is thought to be linked to the greater nutritional demands placed upon females due to their higher maternal physiological investment in reproduction and lactation (Windberg et al. 1991; Pulliainen et al. 1995).

Although season influenced disease prevalence, it did not appear to influence serval BCI. This contrasts with previous research that concluded that seasonal changes had a significant effect on body condition of Eurasian lynx (Pulliainen et al. 1995). This may be because seasonal changes at Secunda are less extreme than in Finland, and prey availability therefore varies to a much smaller extent between the seasons. A similar trend was also observed in coyotes in Texas, although interestingly fat deposits varied between seasons, but not overall body weight (Windberg et al. 1991). It is possible that although BCI did not change between seasons, more subtle metabolic changes such as these would have been missed.

FCoV had the highest prevalence in servals and is also common in caracal (Collier and O'Brien 1985; Thalwitzer et al. 2010). This is particularly important since FCoV has been reported as a source of mortality for servals in captivity (Juan-Sallés et al. 1998). Despite these threats, there are few data regarding coronavirus dynamics in wild populations. While season was shown as a key driver, it is unknown how seasonality might manifest in behaviors that affect infection risk.

## Conclusion

The prevalence of antibodies to the most viruses for which we screened appeared to be high for servals at Secunda, although there are few other studies with which these prevalence can be compared. High viral infection prevalence may be linked to the high population density, season, and sex. We found little evidence that body condition played a large role in viral infection prevalence, and sex was the only factor that influenced body condition, with males in better physical condition than females. We therefore recommend continued viral surveillance of this population and suggest that long-term studies of changes in viral prevalence would be extremely useful to determine which factors influence felid epidemiology on a broader scale.

## Supplementary Information

Below is the link to the electronic supplementary material.

Supplementary file1 (DOCX 386 kb)

## Data Availability

The datasets generated during and/or analyzed during the current study are available in the GitHub repository [https://github.com/lourens-swanepoel/Serval_health].
