# Supplementary material for: Viral Prevalence in Wild Serval Population is Driven by Season and Sex
Source: Ecohealth. 2021 May 31;18(1):113–22. doi: 10.1007/s10393-021-01533-z (PMC8166362; doi:10.1007/s10393-021-01533-z)
Supplement: Supplementary file 1 — Supplementary file1 (DOCX 386 kb) [file 10393_2021_1533_MOESM1_ESM.docx]

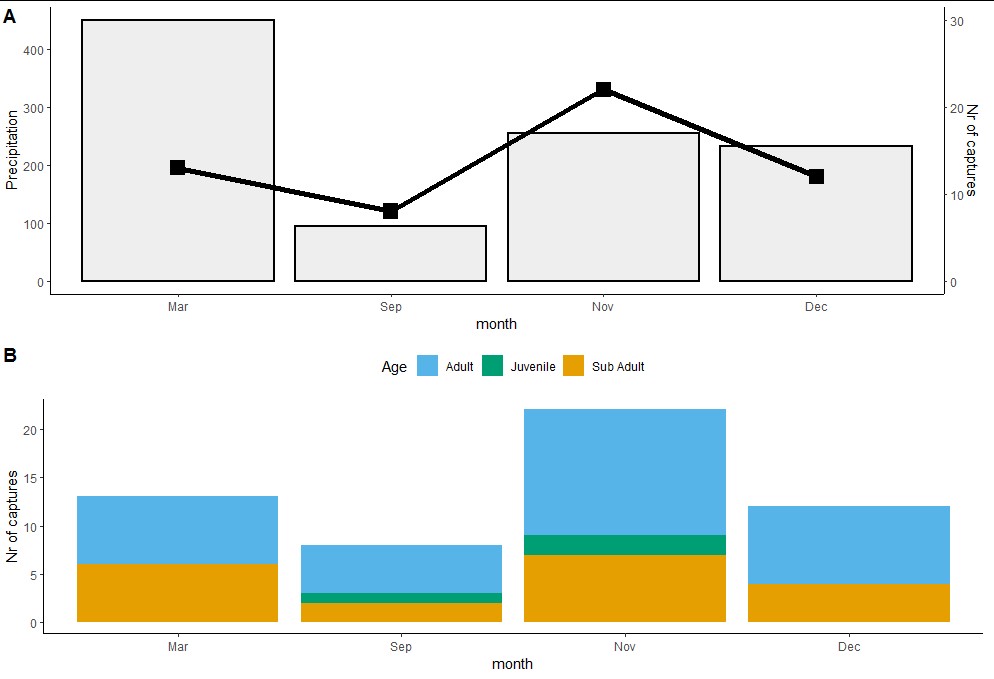


Fig. 1 S. Serval capture rates A) in relation to monthly rainfall and B) capture rates per month per age and sex class.


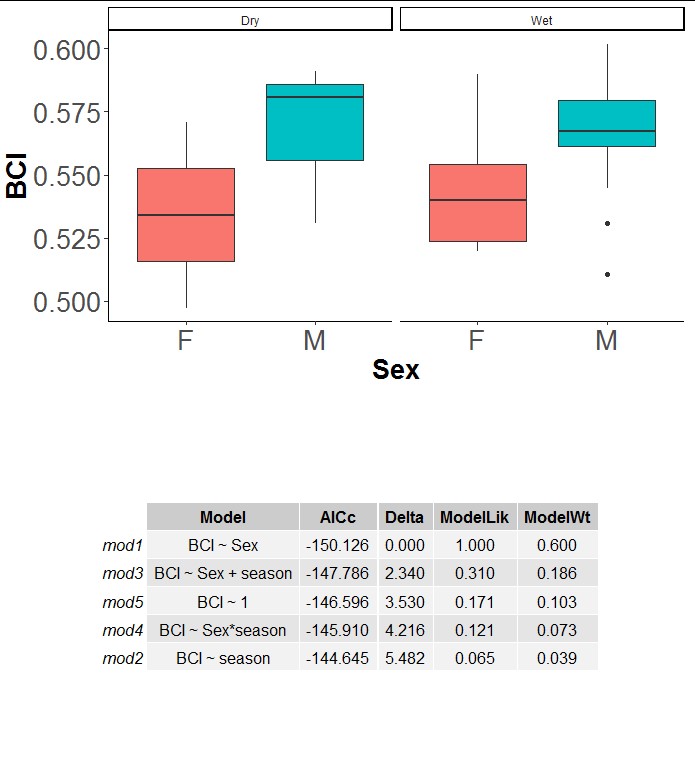


Fig. 2 S. Top) Effect of sex on BCI and, Bottom) model rankings for variables affecting BCI.


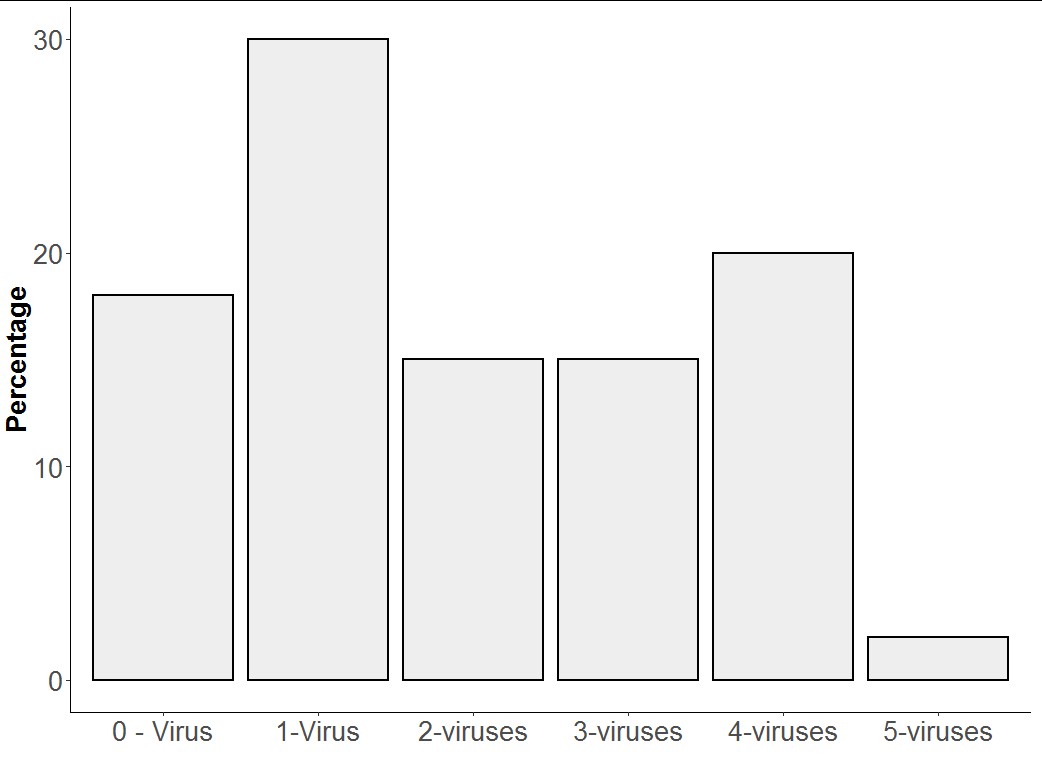


Fig. 3S. Number of serval testing positive for different number of viruses.


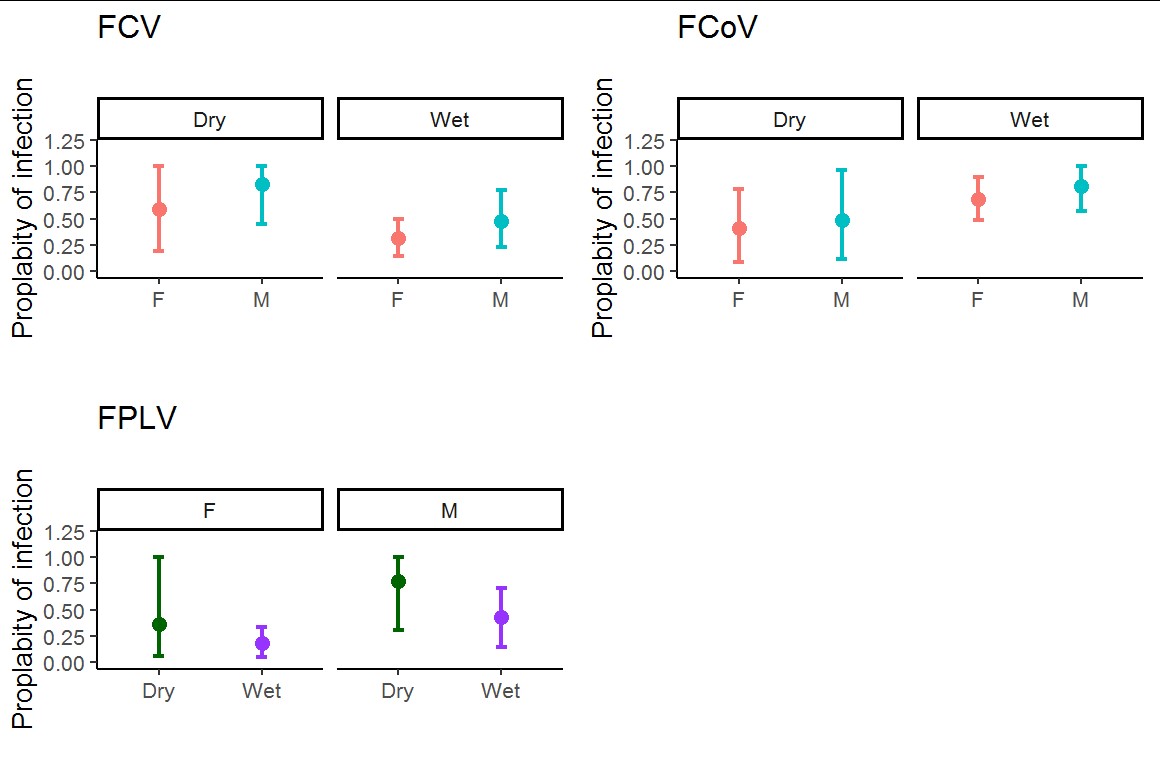


Fig. 4S. Models predicting probability of viral infections for season + sex (FCV and FCoV), showing higher overlap in CI between sexes. Likelihood ratio tests suggested that dropping sex from FCoV (p = 0.465) and FCV (p = 0.0515) improved model fit. FPLV model showing high CI overlap between seasons in probability of infection. Likelihood ratio test suggested that dropping season improved model fit (p = 0.202).


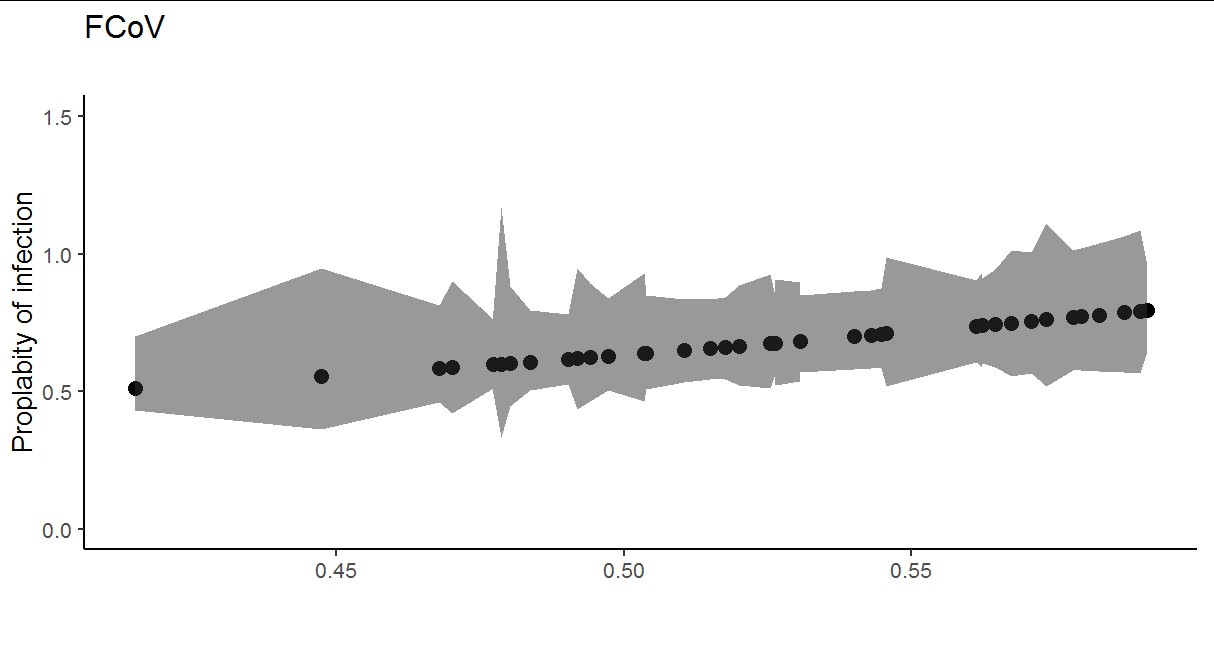


Fig. 5S. Effect of BCI scores on probability of FCoV infection. Grey shading represents 95% confidence interval around predicated point estimates.

Table 1S: The total model results and ranking for the various variables affecting the different disease probability.


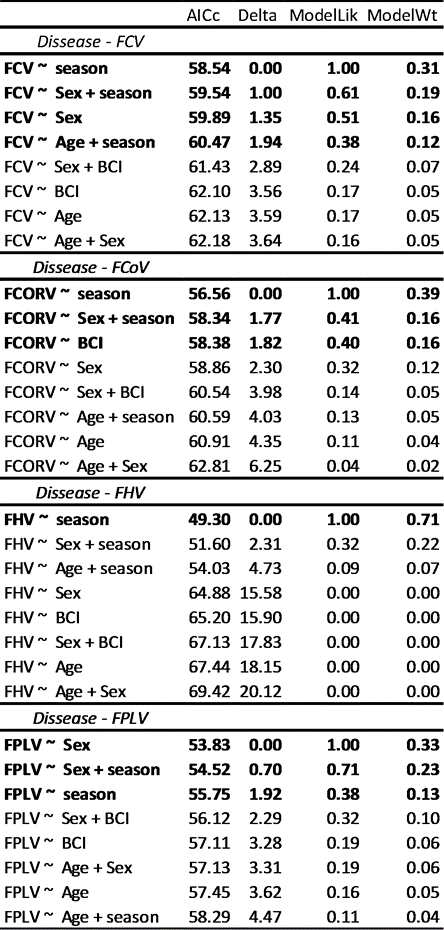


Table 2S. This table contains some of the capture information including all recaptures, indicating the seroprevalence. Additional information collected but not included in this table is the morphological data.
